# Preventive Inoculation for Yellow Fever

**Published:** 1886-06

**Authors:** 


### Preventive Inoculation for Yellow Fever.

In consequence of the severe epidemic of yellow fever which occurred in Brazil in 1883 and 1884, the Emperor, Dom Pedro, appointed Dr. Domingos Freire, Professor of Organic Chemistry and Biology in the College of Medicine of Rio Janeiro, to study the disease. Previous to this, in 1880, Prof. Freire had ascertained that a specific microbe was found in all the tissues and fluids of yellow fever patients, consequently his investigations during the past year have been merely a continuation of previous studies. First, experiments were made to demonstrate the contagiousness of the malady. Guinea pigs and rabbits were successfully inoculated; chickens, pigeons, monkeys and dogs were insusceptible. The inoculation of blood, urine and all the fluids of the body would produce the disease. Water, previously sterilized by heat, and then filtered through muscular or nervous tissue, also became infectious. The rabbits inoculated with any of these fluids contracted yellow fever, and their blood or urine in turn, on inoculation, produced the disease in other animals. By successive inoculations, the virus became milder in its action, and finally failed to produce fatal cases. A microscopic examination of the air in which the patients lived revealed the presence of the specific micro-organisms. These were also found in the water and soil about the infected patients.

Having demonstrated the contagiousness of yellow fever, the existence of a specific micro-organism in the patients and the surrounding media, and its increase in various culture fluids, Dr. Freire observed that the power of the xanthogenic virus was weakened by transmission to animals known to be refractory to its action, such as chickens and pigeons. By inoculating rabbits with the blood or other fluids taken from patients during the mild period of the epidemic in June and July, it was observed that the animals did not die. During the epidemic of last year, Freire made 418 inoculations, only seven of whom died; during the same period 650 uninoculated persons died. Up to the 6th of last October he had inoculated 6,000 individuals. This virus, modified and cultivated, is a safe and certain preventive.

Dr. Issatier, while traveling through Rio Janeiro, was inoculated by Freire. Half a gramme of the infectious fluid was inoculated in the deltoid region at noon. The following symptoms were developed:

At 2.15 P.M., temperature 99.5°, at 4.10 the same, at 6.45 temperature 100.4°, at 8.30 temperature 101.3°, at 9 P.M. the same. There was an increased pulse and frontal headache. He slept well until 5 A. M. These were the only symptoms observed.

The statistics of Freire show a mortality of 1.6 per cent. among the inoculated, while of the uninoculated, who contract the disease, 13.7 per cent die.

In a letter dated October 6th, Freire stated that, while yellow fever was the cause of 300 deaths in Rio Janeiro, in 1885, not one of these deaths occurred among the 5,000 who had been inoculated.—*Revista Medico-Social de Madrid.*

